# Using formative evaluation in an implementation project to increase vaccination rates in high-risk veterans: QUERI Series

**DOI:** 10.1186/1748-5908-3-22

**Published:** 2008-04-22

**Authors:** Carolyn M Wallace, Marcia W Legro

**Affiliations:** 1 Health Services Research & Development, VA Puget Sound Health Care System, Seattle, Washington, USA

## Abstract

**Background:**

Implementation of research into practice in health care systems is a challenging and often unsuccessful endeavor. The United States Department of Veterans Affairs (VA) Quality Enhancement Research Initiative (QUERI) research teams include formative evaluations (FE) in their action-oriented VA implementation projects to identify critical information about the processes of implementation that can guide adjustments to project activities, in order to better meet project goals. This article describes the development and use of FE in an action-oriented implementation research project.

**Methods:**

This two-year action-oriented implementation research project was conducted at 23 VA Spinal Cord Injury (SCI) Centers, and targeted patients, staff and the system of care, such as administration and information technology. Data for FE were collected by electronic and paper surveys, semi-structured and open-ended interviews, notes during conference calls, and exchange of e-mail messages. Specific questions were developed for each intervention (designed to improve vaccination rates for influenza in veterans with spinal cord injury and disorder); informants were selected for their knowledge of interventions and their use in SCI Centers.

**Results:**

Data from FE were compiled separately for each intervention to describe barriers to progress and guide adjustments to implementation activities. These data addressed the processes of implementing the interventions, problem-solving activities and the status of interventions at SCI Centers.

**Conclusion:**

Formative evaluations provided the project team with a broad view of the processes of implementing multi-targeted interventions as well as the evolving status of the related best practice. Using FE was useful, although the challenges of conducting FE for non-field researchers should be addressed. Work is needed to develop methods for conducting FE across multiple sites, as well as acknowledging variations in local contexts that affect implementation of interventions.

## Background

Implementation of research into practice in health care systems is a challenging and often unsuccessful endeavor, particularly when those persons introducing or researching change fail to adequately understand and modify the context and progress of implementation or make appropriate adjustments to achieve goals. Formative evaluation (FE) – a long-standing technique in program evaluation – can play an important part in implementation projects. Using FE can provide critical information about the processes of implementation that can enhance the success and understanding of projects designed to improve health care.

This article is one in a *Series *of articles documenting implementation science frameworks and approaches developed by the U.S. Department of Veterans Affairs (VA) Quality Enhancement Research Initiative (QUERI). QUERI is briefly outlined in Table 1 and described in more detail in previous publications [1,2]. The *Series' *introductory article [3] highlights aspects of QUERI that are related specifically to implementation science, and describes additional types of articles contained in the *QUERI Series*.

**Table 1:** The VA Quality Enhancement Research Initiative (QUERI)

The U.S. Department of Veterans Affairs' (VA) Quality Enhancement Research Initiative (QUERI) was launched in 1998. QUERI was designed to harness VA's health services research expertise and resources in an ongoing system-wide effort to improve the performance of the VA healthcare system and, thus, quality of care for veterans.
QUERI researchers collaborate with VA policy and practice leaders, clinicians, and operations staff to implement appropriate evidence-based practices into routine clinical care. They work within distinct disease- or condition-specific QUERI Centers and utilize a standard six-step process:
1) Identify high-risk/high-volume diseases or problems.
2) Identify best practices.
3) Define existing practice patterns and outcomes across the VA and current variation from best practices.
4) Identify and implement interventions to promote best practices.
5) Document that best practices improve outcomes.
6) Document that outcomes are associated with improved health-related quality of life.
Within Step 4, QUERI implementation efforts generally follow a sequence of four phases to enable the refinement and spread of effective and sustainable implementation programs across multiple VA medical centers and clinics. The phases include:
1) Single site pilot,
2) Small scale, multi-site implementation trial,
3) Large scale, multi-region implementation trial, and
4) System-wide rollout.
Researchers employ additional QUERI frameworks and tools, as highlighted in this *Series*, to enhance achievement of each project's quality improvement and implementation science goals.

The implementation research project was developed by the SCI-QUERI group, which used the QUERI 6-step framework to establish priorities for its work [4]. Using a repeated measures quality improvement design, this project had two purposes: 1) to improve the vaccination rate for influenza in veterans with a spinal cord injury and disorder (SCI&D), and 2) to oversee the process of implementing several integrated, evidence-based interventions selected to enhance adoption of the targeted best clinical practice. The two-year project involved 23 VA SCI Centers that provide primary and specialty care to veterans with SCI&D.

The main outcome measure for the summative evaluation was the rate for annual influenza vaccination in veterans with SCI&D, based on patient self-reported influenza vaccination status. The summative evaluation for this implementation project is described elsewhere [5]. The second purpose of the project, and the specific focus of this paper, was the use of FE both to monitor and enhance the processes of implementing multi-targeted interventions in the SCI Centers [6]. This project received human subjects approval at the Hines VA Medical Center and the University of Washington, for the VA Puget Sound Health Care System.

Although FE was not unique to VA QUERI projects, it was important to this project (and to the QUERI approach) because it can illuminate the processes that facilitate or impede progress in implementation research. The project team used a working definition of FE throughout the project to focus on monitoring, describing and refining the process of implementation. Although FE and its underlying ideas were discussed among members of QUERI groups and highlighted by QUERI leadership and experts [3], the specific stages of FE had not been articulated as such during the time this project was conducted. The article by Stetler et al. on formative evaluation had not yet been published [6], so it did not serve as a guide to FE during this project. Nonetheless, its concepts reflect the general purposes of FE in this implementation research project: i.e., to identify and describe pre-existing and emergent barriers for each intervention, to obtain sufficient information to enable the team to address identified barriers, and to assess the progress in operationalizing the interventions [6].

In summary, this article describes the application of FE processes and practices in this project, including how FE was developed and carried out; barriers to and facilitators of FE; application of results of FE to refine implementation activities; and how FE was affected by the characteristics of the project. We also will discuss the strengths and weaknesses of our FE approach and activities, measurement issues, organization and presentation of FE data and results, and designing FE.

## Methods

### Description of interventions

Four interventions were selected for implementation during this project, based on literature review and the applicability of the proposed interventions to the SCI Centers. Table 2 provides an overview of the interventions that were directed at *patients*, *providers *involved in vaccine delivery, and the *health care system*. The interventions were: reminder letters and education materials for patients, educational materials for providers, use of the computerized clinical reminder (CCR) for influenza, and standing orders (for nurses to screen and offer vaccines without an order). This article will address FE that was conducted on reminder letters to patients, use of the CCR for influenza, and standing orders. It should be noted that an implementation intervention used in this project to enhance adoption of the clinical and delivery system interventions was facilitation, which is described elsewhere [3,7].

**Table 2:** Overview of interventions

Implementation Intervention	Directed at	When used in project	Formative evaluations
Reminder letters and information	Patients with SCI&D	September/October of 1^st ^and 2^nd ^years of project (beginning of influenza season)	1^st ^and 2^nd ^years of project
CCR for influenza vaccine	Health care system (electronic medical record)	CCR was installed in medical record system prior to project	1^st ^and 2^nd ^years of project
Standing orders	Health care system	Variable, depending on circumstances at each VAMC	Ongoing during 2^nd ^year of project

The interventions were presented to staff at the SCI Centers at the beginning of the project via announcements at the monthly SCI Chiefs conference call and a short presentation at a conference for the administrative officers of the SCI Centers. The project team described the interventions as a means for them to reach the newly established SCI performance measures for rates of influenza and pneumonia vaccinations. Although there was no requirement to adopt and implement the interventions, staff at the SCI Centers were aware of the expectation to achieve the performance measure target rates for vaccinations. However, it was the project team's implicit goal that these interventions become routine practices to the extent possible.

### Overview of formative evaluations

A broad base of formative evidence was collected in order to describe and understand the context in which the interventions were implemented in each of 23 SCI Centers [8]. Two members of the implementation project team (ML and CW) carried out FE. Prior to conducting any FE activities, they clarified specific objectives for each intervention, formulated evaluative questions, developed semi-structured interviews tailored to each intervention, and identified informants. (See Table 3 for an overview of FE, with examples of questions and responses.)

**Table 3:** Overview of formative evaluations

Implementation Intervention	Purpose of implementation intervention	Examples of FE questions	Examples of FE data and their use
Reminder letters	Information to patients	What information did staff at SCI Centers need to have to prepare and mail letters?	Specific information was added to the general letter; staff could make a patient list and prepare labels.
		Could staff prepare and mail letters without assistance from project team?	Staff called on project team for help with letters or labels.
Use of CCR for influenza	Document vaccination status of patients	To IT staff: What version of this CCR is installed at your VAMC?	CCR version # verified as correct one (most recent one) for use
		To others: How do you document that a patient was screened and received influenza vaccine?	
			We use the CCR for influenza.
		Can you use the CCR for influenza for all patients?	We use another template in the electronic medical record.
			Yes; we use it for all our patients, including home care patients and those who got a 'flu shot' outside VA.
			No; we can't use it for inpatients.
		Do all staff who take care of patients have access to the CCR for influenza?	Yes.
			No; access by some nurses is restricted by the VAMC.
Standing Orders	Nurses allowed to screen and offer vaccine to patients without a specific order.	Is there a standing orders policy in your SCI Center?	Yes.
			No; the VAMC does not allow standing orders.
			We have a protocol for influenza vaccinations that allows nurses to screen patients and offer the vaccine.

A semi-structured interview was conducted for each intervention, via telephone calls with staff in SCI Centers or other departments. Informants were selected for their knowledge and ability to provide detailed information about a specific intervention in an SCI Center and its associated medical center and their willingness to answer questions [9]. More than one informant was interviewed for the CCR for influenza and standing orders interventions. [Standing orders (for this project): a protocol or a limited general order for influenza vaccine.] Different informants were identified for each of the interventions because knowledge about each intervention and its use in a SCI Center was required. Data from interviews were transcribed and entered into tables. Summary tables were prepared for specific questions, and notes from interviews were retained in separate files.

The project team also held periodic conference calls to discuss the interventions. Participation in these calls was voluntary and included clinical staff and administrators from SCI Centers, the project team members who conducted FE, and the principal investigators for the project. Notes were taken during the conference call by a project team member (CW). These notes were put into transcript form and reviewed by team members who participated in the call. Notes from conference calls were retained. During the project, the team also sent a one-page electronic newsletter to SCI Centers with specific information about interventions or outcomes data, for example. A project team member (CW) used 1:1 telephone calls to discuss specific issues, provide information, or to answer questions as they arose during the project.

### Carrying out formative evaluations

#### Reminder letter and information

The plan for FE for this intervention was to assess the ability of staff at each SCI Center to carry out all activities for this intervention through a two-step, two-year process. FE helped to address optimal use of this intervention by identifying 1) barriers to the following preparatory activities for Year 1 and 2) related feasibility issues for Year 2, when staff were envisioned to take on routine implementation. Activities for Year 1 included preparation of an electronic file of patient addresses from a registry maintained by staff at each SCI Center; formatting the addresses for mailing labels; modification of a standard letter to be sent to patients with SCI&D to include when and where the vaccine would be available at the SCI Center or the associated hospital; and inclusion of signatures of clinical staff familiar to patients.

FE data for Year 1 were collected throughout the process of preparing and mailing the materials, with questions that addressed the capability of staff at each SCI Center to carry out each part of the intervention. In addition, proxy data for capability included the time period between when the patient list was requested from each SCI Center and received by the project team, any assistance required to generate the patient list, dates the standard letter was sent to SCI Centers and dates a specific version of the letter was received by the project team, and dates the letters and flyers were mailed to patients.

For Year 2, FE focused on the project team's request that staff at SCI Centers take over the preparation and mailing of patient letters and materials. FE data included 'yes' or 'no' from SCI Centers about preparing and mailing letters and materials to patients, requests for assistance, advice about the process or materials and/or specific assistance provided by the project team.

#### Use of CCR for influenza

The project team focused on ensuring use of the CCR for influenza by staff in SCI Centers because this CCR was developed nationally and installed by staff at each VA medical center (VAMC) prior to the implementation project. The purposes of formative evaluation for the CCR for influenza were to identify barriers and explore contextual factors related to its use in SCI Centers. Data collection addressed: access by staff to the CCR for influenza, availability of technical support in the SCI Center and from VAMC information technology (IT) staff, and use of the CCR for documentation of vaccine receipt.

Several rounds of FE were conducted. The first round was an electronic survey of information technology personnel to verify that the most current version of the CCR for influenza was installed at each VAMC with an associated SCI Center. A second round of FE was a conference call, in which participants identified a variety of problems: inaccurate identification of patients with SCI&D by the CCR for influenza, inconvenient or difficult access to the CCR in the electronic medical record, and use of the CCR limited to particular clinical staff (sometimes excluding nurses). A third evaluation of the CCR for influenza used semi-structured interviews with nurses in the SCI Centers about use of this CCR for inpatients, outpatients, and home care patients. Follow-up interviews were used to track progress in addressing barriers and for further problem-solving.

#### Standing orders for influenza vaccine

A standing orders policy authorizes nurses to screen patients and administer influenza vaccine without a specific order for each patient. The purposes of FE were to assess: the status of a standing orders policy in SCI Centers and associated medical centers, knowledge about standing orders, and policies and practices for influenza vaccine at each SCI Center. The project team planned to provide information about establishing standing orders, or to address any barriers to their use in the SCI Centers.

## Results

### Reminder letter and information

FE data from Year 1 were used immediately to provide staff at SCI Centers with specific assistance to generate the lists of patients. The project team also identified data management problems at some SCI Centers that led to difficulties in formatting mailing labels. The project team reviewed drafts of customized letters to ensure that information such as influenza vaccine clinics was added to the standard letter. For year 2, the project team received reports from staff at 19 of 23 SCI Centers reporting their willingness to take over this intervention. (See Table 4)

**Table 4:** Results of formative evaluations

Intervention	Status at project completion	Comments
Letters to patients	Staff at 21 of 23 SCI Centers mailed letters to patients.	Project team sent a reminder to staff at SCI Centers about letters to patients prior to 2^nd ^year of project. We also asked if staff wanted to prepare and mail letters without our help.
CCR for influenza	The CCR for influenza was used at 16 SCI Centers; another 2 SCI Centers used another template in the electronic medical record; status of use of the CCR for influenza was unknown at 5 SCI Centers.	Variation in use of CCR for influenza was documented by FE.
Standing orders	Standing orders, a protocol or a limited general order for influenza vaccine *for outpatients only*, were in place at 15 SCI Centers. No standing orders, protocol, or limited order were in place at 4 SCI Centers; unknown at 4 SCI Centers.	Some VAMCs did not have a standing orders policy, but used a protocol or a time-limited general order for influenza vaccine.

### Use of CCR for influenza

Analysis of the FE data from the survey of VAMCs showed that the CCR for influenza did not identify all veterans with SCI&D. Further investigation revealed an incomplete list of codes in the taxonomy used by the CCR to identify patients. A complete list of ICD-9 codes to identify these veterans was developed and distributed to IT staff at VAMCs with an associated SCI Center. When the taxonomy for the CCR was revised by the addition of these codes, the CCR would accurately identify all patients with SCI&D.

Another FE – a conference call about the CCR for influenza with staff from SCI Centers – identified barriers to using the CCR to document influenza vaccinations. These data led the team to learn more about the components of the CCR for influenza, other locations to document vaccinations in the electronic medical record, and advantages (and disadvantages) of those methods. The team then recommended use of the CCR for influenza to document receipt of influenza vaccine (in VA or outside VA), refusal of vaccine or vaccine not offered, thereby creating a vaccination history for patients.

The project team also benefited from this approach to FE through the identification of problems that the team had not anticipated, but needed to address in order to enhance implementation of the interventions. For example, although the project team had expected that the nationally-developed and distributed CCR for influenza would be used at all SCI Centers, we did not anticipate the variation in access to this CCR, variation in availability of IT support to SCI Center staff, nor other barriers to its use that we found through FE. Barriers included inability to use the CCR for influenza for inpatients, insufficient training and technical support for staff, and decisions by IT staff about the CCR that made access to it cumbersome and time-consuming for providers. We have discussed limited access by nurses to the CCR for influenza in detail in another article [10]. (See Table 4)

### Results of FE for standing orders

FE data for standing orders also revealed unanticipated variation. We found several mechanisms besides standing orders that authorized nurses to screen and offer influenza vaccine to outpatients without an order – a protocol, blanket order or procedure. The team member conducting FE interviews found that using the term "standing orders" often resulted in a question from informants. When the interviewer asked a general question, "Can nurses screen patients and offer influenza vaccine without an order from a provider?" informants provided information describing various mechanisms for nurses to screen and vaccinate patients. Analysis of FE data also revealed differences in the applicability of standing orders for inpatients, outpatients, and home care patients. For two SCI Centers that did not have a standing orders policy in place, a team member provided examples of standing orders policies. Follow-up interviews found that a standing orders policy was under development at one SCI Center and under discussion at the other. (See Table 4)

## Discussion

Formative evaluations were used in this project to address the processes of implementing and enhancing adoption of multi-targeted interventions selected to increase vaccination rates for respiratory illnesses in veterans with SCI&D. We did not design FE prospectively, but focused instead on emergent issues and follow-up to those issues. The project team used FE to understand contextual and organizational issues in VA Medical Centers (with associated SCI Centers), as well as to describe specific problems with interventions and to address barriers to their implementation in SCI Centers.

In this project, the strengths of the FE were that it was linked to each specific intervention, responded to issues as they arose, focused on processes and addressed the context of the interventions. However, the authors of this paper also found an inadequate estimate of the time and resources necessary to collect, analyze and use FE data. In addition, the team found unexpected variation and complexity in implementation processes and status of interventions, in part, because FE was not designed prospectively.

The FE activities for this project followed the two-year timeline for the research component of this project and the timing of influenza vaccinations. Although this project did not place researchers 'in the field,' the team introduced itself and the project to staff at the SCI Centers prior to the optimal time period to receive influenza vaccine, and maintained contact with the sites about the project. The introduction to the sites and ongoing connection with staff at sites were important parts of the project. Team members were aware of some limitations because we had no presence "in the field;" no observational data to use to verify FE data collected in other ways; limitations to team members' understanding of local context; and unfamiliarity of staff at SCI Centers with project team members, and of team members with them. We addressed our non-field presence with: conference calls, 1:1 calls for information-gathering, problem-solving and follow-up activities, an electronic newsletter, and reports at the monthly SCI chiefs' call.

The project team's work depended on and was assisted by the willingness of staff in SCI Centers to participate in both formative and summative data collection activities, and to answer team members' questions. The team conducted FE to address the goals of the project, while not burdening the relatively small staffs at SCI Centers with FE activities (conference calls, e-mail messages, and 1:1 interviews). The team also recognized a particular factor in our problem-solving and assistance to staff at SCI Centers – as team members, we were from "outside," because we had no staff in the field. As a result, we relied on descriptions of problems and as many telephone calls and e-mail messages as needed to address problems. Since the project team had no authority to order the implementation of interventions, we often made general rather than specific suggestions. This collaboration in identifying problems and proposing solutions for staff in SCI Centers was an important component of the "outside" approach.

FE data collection in this project was guided by the quality improvement component of the project – increasing vaccination rates for influenza among veterans with spinal cord injury and disorder. The general descriptions of the interventions provided the basis for FE questions as well as the processes to be expected in putting them into place. The project team used information from conference calls, whenever possible, to inform the development of semi-structured interviews about each intervention. When informants answered questions and provided information during interviews, the interviewer followed up in order to clarify responses and to determine additional information to be gathered from other sources.

Analysis of the FE data focused on monitoring and describing the processes of implementing the interventions. The team was especially interested in identifying problems and describing them so that issues related to the organizational context, processes of care, availability of resources, and which staff were involved could be addressed. We found that respondents to FE questions could sometimes not only describe problems, but contribute to understanding the sources of the problems as well. The team focused on identifying problems and/or issues in ways that made sense to staff in SCI Centers, so that they could participate in addressing those problems or issues. The team found that general suggestions were appropriate for some issues, such as the need for IT support and/or training in using the CCR for influenza. When more information or specific information was needed, the team could provide suggestions about appropriate personnel to contact. The team also used different respondents in order to have a wide range of perspectives represented, particularly when an intervention addressed different levels of the organization.

The project team's approach to the use of FE data during the project was to conduct the best evaluation possible, and to make suggestions for adjustments to the implementation processes based on the analyses of the available data. The next steps required: flexibility and persistence, an iterative process of selecting and applying suggestions, making adjustments to local circumstances, and evaluating the results. Although FE data may be used to modify interventions or to make changes to their delivery, the purpose of FE in this project was to enhance implementation, not to maintain a prescribed method of delivery.

The team encountered a measurement issue that was important for the research component of the implementation project. The approach to tailoring use of the interventions at SCI Centers meant that it became difficult to describe and interpret the status of each intervention, in terms of a standard measure of "integrity" or "fidelity" for each intervention that would allow comparisons across Centers. For example, the team found that some differences among SCI Centers in implementation of interventions existed because of autonomy of VAMCs, decentralized decision-making, and local policies. These were factors which neither the project team nor staff at SCI Centers could address.

The study design included a system to quantify the qualitative data about the status of each intervention at SCI Centers. Once quantified, this data would have been used in a multivariate analysis of the overall project. However, the variation due to contextual factors or local activities meant that the complex questions did not apply. The team attempted to address this problem by assigning multiple scores to detailed questions about the status of the interventions at each SCI Center, but this proposed solution failed. The team then developed a less complex scoring system about the operational status of each intervention. This scoring system did not produce much variation across SCI Centers and, therefore, was not useful in the final multivariate analysis.

As we (ML and CW) conducted FE activities during the project, we prepared reports on these activities for the full project team. These reports focused on the status of interventions at SCI Centers, overview of results of FE, and planned activities by the project team. Although these reports were useful, we found it difficult to describe the status of interventions by 'yes' or 'no,' or other short responses, and to briefly characterize follow-up activities. Planning FE activities prospectively to include reports of implementation status of each intervention (e.g., 'Is the intervention 'in place?') and implementation processes (e.g., 'What's happening?') could be informative.

## Conclusion

FE was an important component of this project because FE activities allowed the project team to have a broad view of the processes of implementing the evidence-based interventions selected to achieve the outcomes goal of this project – improvement in vaccination rates for influenza vaccine among veterans with spinal cord injury and disorder. At the same time, these evaluations provided the project team with information about barriers to implementation that guided problem-solving activities and helped the implementation team refine its assistance to staff in SCI Centers.

Having completed the project and reviewed the formative evaluations conducted during the project, we think that FE conducted during the project can be best understood as developmental FE and implementation-focused FE [6]. These evaluations, or assessments of implementation processes, occurred at different stages of the implementation project. Developmental FE, a diagnostic analysis, occurred during the first stage of the implementation of each intervention. Implementation-focused FE focused on actual implementation processes, the influences on these processes and barriers to implementation.

We did not use the terms developmental FE or implementation-focused FE, although we think they can provide a useful guide for implementation researchers by focusing FE activities and clarifying their purposes in projects. Future implementation projects should report FE findings, whether projects are sponsored by VA QUERI or other sources. Development of measurement and analytic methods for conducting FE at multiple sites, while accounting for local contexts, would be particularly useful. Methods of conducting FE for non-field researchers also need to be addressed so that FE can be usefully employed in health care systems with geographically dispersed facilities.

We will not address the issue of intervention fidelity in this paper, although it is an important consideration for implementation projects, particularly for reporting results. Although the project team conducted surveys of veterans with SCI&D to ask about their receipt of influenza vaccine, we did not explicitly use these results for progress-focused FE. We also did not conduct interpretive FE for this project. Interpretive FE, using data from other FE activities to further explain the processes and outcomes of implementation activities, follows the active stages of implementing interventions during a project [6]. Although conducting such analyses could provide additional information about implementation processes, the design and conduct of these analyses need to be carefully considered so they benefit and inform subsequent projects, as well as the field of implementation research.

## List of abbreviations used

CCR: Computerized Clinical Reminder; FE: Formative Evaluation; IT: Information Technology; QUERI: Quality Enhancement Research Initiative; SCI: Spinal Cord Injury; SCI&D: Spinal Cord Injury and Disorder; VA: U.S. Department of Veterans Affairs; VAMC: Veterans Affairs Medical Center.

## Competing interests

The authors declare that they have no competing interests.

## Authors' contributions

ML participated in the conception and design of the research project from which data for this manuscript was acquired. Both authors (ML and CW) participated in the conception and design of the formative evaluation component of the project, including acquisition, analysis and interpretation of formative evaluation data. CW drafted the manuscript; both authors have participated in revisions for important intellectual content. ML and CW have given final approval of the version of the manuscript to be published.
